# Associations of social engagement and loneliness with the progression and reversal of frailty: longitudinal investigations of 2 prospective cohorts from the UK and the USA

**DOI:** 10.1093/aje/kwae221

**Published:** 2024-07-25

**Authors:** Ziyi Cai, Anna Olia Papacosta, Lucy T Lennon, Peter H Whincup, Sasiwarang Goya Wannamethee, Eleanor M Simonsick, John C Mathers, Sheena E Ramsay

**Affiliations:** Population Health Sciences Institute, Newcastle University, Newcastle upon Tyne, United Kingdom; Department of Primary Care and Population Health, UCL, London, United Kingdom; Department of Primary Care and Population Health, UCL, London, United Kingdom; Population Health Research Institute, St George's, University of London, Cranmer Terrace, London, United Kingdom; Department of Primary Care and Population Health, UCL, London, United Kingdom; National Institute on Aging Intramural Research Program, NIH, Baltimore, MD, United States; Population Health Sciences Institute, Newcastle University, Newcastle upon Tyne, United Kingdom; Population Health Sciences Institute, Newcastle University, Newcastle upon Tyne, United Kingdom

**Keywords:** frailty, social engagement, loneliness, social connections, cohort study, aging

## Abstract

Social connections may impact the dynamic trajectory of frailty. Using data from the British Regional Heart Study (BRHS) in the UK (*n* = 715) and the US Health, Aging and Body Composition (Health ABC) Study (*n* = 1256), we conducted multinominal regression analyses to examine the association of baseline and change in social engagement and loneliness with progression to prefrailty and frailty, as well as their association with reversal to prefrailty and robust status among older adults. A higher level of social engagement at baseline (BRHS: relative risk ratio [RRR] 0.69 [95% CI, 0.55–0.85]; Health ABC: 0.56 [0.45-0.70]) and an increase in social engagement (BRHS: 0.73 [0.59-0.90]; Health ABC: 0.51 [0.41-0.63]) were associated with a lower risk of developing frailty. In BRHS, a higher level of loneliness at baseline (1.42 [1.10-1.83]) and an increase in loneliness (1.50 [1.18-1.90]) raised the risk of developing frailty. For reversal of frailty, higher social engagement at baseline (Health ABC: 1.63 [1.08-2.47]) and an increase in social engagement (BRHS: 1.74 [1.18-2.50]; Health ABC: 1.79 [1.17-.274]) were beneficial. Social connections may be potentially important and modifiable factors in both preventing and reversing progression of frailty in older adults.

## Introduction

Increased life expectancy has contributed to an aging population globally. Frailty, a complex age-related syndrome characterized by a cumulative deficit in many physiological systems and heightened vulnerability to stressors, is common among older adults.^1^ An estimated 10% of older adults aged over 65 years are frail^2^ and at higher risk of falls, disability, hospitalization, long-term care, and death.^3^^-^^7^ With the rapid expansion of an aging population, the proportion of frail individuals has increased over time, which places a substantial burden on the health and social care systems.^8^ However, frailty is not inevitable. A proportion (up to three-fourths) of people over 85 years old remain nonfrail.^1^ Moreover, as some individuals can recover from frailty,^9^ identifying factors that contribute to the reversal of frailty is also important.

Measures of frailty have been developed for clinical assessment in health and social care settings. A landmark study by Fried et al.^10^ proposes a frailty phenotype model that assesses physical frailty through 5 criteria: unintentional weight loss, weakness or poor handgrip strength, exhaustion, slow walking speed, and low physical activity. Another measure frailty index proposed by Mitnitski et al.^11^ is based on the cumulative deficit model that assesses frailty by a long checklist of clinical conditions and disease. Although these 2 measures have been extensively validated and are widely used for assessing frailty, they are built based on different concepts and serve different purposes. The frailty phenotype is more suitable for initial stratification of the population to different frailty profiles, while the frailty index summarizes the results of comprehensive geriatric assessment and acts as an objective marker of deficits accumulation.^12^

Social connections, including quantitative (ie, levels of social engagement) and qualitative (ie, loneliness) aspects, have been theorized to contribute to a wide range of health outcomes, including frailty.^13^ The concept of social engagement focuses on the structural aspects, and it refers to the degree of participation in a community or society.^14^ Social network theory posits that participating in a wider range of social activities could promote health via reinforcing meaningful social roles and providing opportunities for individuals with companionship and sociability.^15^ Loneliness, on the other hand, emphasizes the quality of social interactions. It is a perceived negative feeling associated with the absence of social contacts.^16^ Individuals can feel lonely even if they have participated in extensive social activities; conversely, individuals with low social engagement could be satisfied with the quality of their social relationships.^17^ Theory of loneliness posits that feeling lonely is tantamount to feeling unsafe since humans are a social species. This implicit hypervigilance for social threat in the environment can increase psychological stress, activating neurobiological (eg, elevating sympathetic tone that is responsible for the maintenance of hypertension) and behavioral (eg, diminishing capacity for self-regulation) mechanisms that contribute to adverse health outcomes.^18^ Empirical studies have shown that poor social engagement and/or feeling lonely are significantly associated with increased mortality and morbidity, including cardiovascular disease, cancer, disability, depression, dementia, and cognitive decline.^15^^,^^19^^-^^25^

Previous studies have suggested that these social connections are also linked with frailty in older adults. A study^26^ reported that the risk of developing frailty for people with high levels of social isolation is 30% greater than for those with a low level of social isolation. Similarly, people with a high level of loneliness are around 2.6 times more likely to develop frailty compared with those with lower levels of loneliness. These associations between social connections and frailty have been investigated both cross-sectionally^27^^-^^29^ and longitudinally.^9^^,^^26^^,^^30^^,^^31^ However, very few studies have considered the dynamic nature of an individual’s social engagement, loneliness, and frailty status (ie, changes in these factors over time). Specifically, most studies have focused on healthy individuals and investigated how social engagement and loneliness were linked to the development of frailty.^26^^,^^30^^-^^32^ It remains unclear whether social engagement and loneliness could play a role in altering the frailty status of individuals who are already frail. In addition, most studies assessed social engagement and loneliness as time-invariant factors.^9^^,^^30^^,^^31^ Whether change in social engagement and loneliness influences frailty status over time is underinvestigated. The current study examines the dynamic trajectories of frailty status among community-dwelling older adults. The study is based on 2 population-based cohort studies of older adults in the United States and the United Kingdom, which allows assessing the validity, consistency, and robustness of the associations in 2 population study samples in Western countries. We also aim to examine the research questions regarding whether the baseline and changes in social engagement and loneliness affect the transition of frailty status among older adults. In summary, the questions we examined were whether lower social engagement and higher loneliness are associated with progression to prefrailty and frailty, as well as whether higher social engagement and lower loneliness are associated with reversal of frailty.

## Methods

### Study design and participants

Data from the British Regional Heart Study (BRHS) collected in the United Kingdom and the Health, Aging and Body Composition (Health ABC) Study in the United States were used in this longitudinal study. These are both complementary population-based samples of community-dwelling older adults with comparable measures and follow-up. Examining the associations in these 2 studies allowed the consistency and reproducibility of the associations to be tested in 2 different cohort studies.

The BRHS is an ongoing cohort study established in 1978-1980, including a socially and geographically representative population of 7735 British men aged 40 to 59 years from 24 towns in the United Kingdom.^33^ In the analysis, baseline measures were based on data from the BRHS physical examination and questionnaires in 2010-2012; follow-up measures were from BRHS data collected in 2018. In 2010-2012, 2147 men aged 71 to 92 years attended the study (722 attended physical examinations and 2137 completed questionnaires). Since the questionnaires in 2010-2012 did not include questions on loneliness, we used the data in 2014 as a baseline. A total of 1013 men attended the follow-up study in 2018 (667 attended the follow-up examinations, and 1009 completed the questionnaires).

The Health ABC is a prospective cohort study established in the United States in 1997-1998, with the study population consisting of 3075 White and African American men and women aged 70 to 79 years. White participants were identified from a random sample of Medicare beneficiaries who lived in designated zip code areas surrounding Memphis and Pittsburgh, whereas African American participants were recruited from all age-eligible residents in these zip codes.^34^ Physical assessment and questionnaires in 2002-2003 for participants aged 73 to 85 years served as a baseline for the current analysis, and data collected in 2006-2007 serve as follow-up measures.

### Frailty

Measure of frailty status in both the BRHS and Health ABC cohort studies was determined using the Fried frailty phenotype. Details on the measures of frailty in both studies have been fully described elsewhere^35^ and can be found in Table S1. Briefly, the measure comprised 5 components, including unintentional weight loss, exhaustion, weakness, low physical activity, and slowness. Participants with none of the components were defined as robust, 1 or 2 components as prefrail, and 3 or more as frail.

### Social engagement

In both the BRHS and Health ABC studies, social engagement measures were conceptually similar and based on whether participants engaged in the following social activities in a typical week: (1) spending time with family, friends, and neighbors; (2) doing paid work; (3) doing voluntary work; (4) playing cards, games, or bingo; (5) participating in religious activities or social clubs; (6) going on holidays or overnight trips; (7) reading books or newspapers; (8) using the Internet or writing letters; (9) attending courses or public meetings; and (10) eating out in the restaurants or vising the cinema, sports events, museum, and so on. In the BRHS, participants were asked if they engaged in these social activities with a yes/no response. In the Health ABC, participants were asked about the frequency of these engagements in a typical week, and we recoded “less than once a week” as a “no” response. Detailed information on the questions used for social engagement and corresponding coding can be found in Table S2. Scores on social engagement ranged from 0 to 10, with higher scores indicating a higher level of social engagement. We calculated the change in social engagement as the score at follow-up minus the score at baseline—a positive value indicating an increase in social engagement and a negative value indicating a decrease.

### Loneliness

In the BRHS, subjective perception of loneliness was measured through 4 questions: “how often do you feel you lack companionship?”; “how often do you feel isolated from others?”; “how often do you feel out?”; “how often do you feel in tune with the people around you?” The response options were (1) hardly ever or ever, (2) sometimes, or (3) often. A score of loneliness was according to the sum of all items, which ranged from 0 to 8.

In Health ABC, subjective feeling of loneliness was measured by a single question: “I felt lonely (rarely/none, sometimes, much of the time; most/all of the time”), with a score ranging from 0 to 3. In both studies, higher scores of loneliness indicate greater loneliness. Similar to change in social engagement, change in loneliness was calculated by the score at follow-up minus the score at baseline. A positive value means an increase, while a negative value indicates a decrease in loneliness. Detailed information on measures of loneliness can be found in Table S3.

### Baseline covariates

Information related to sociodemographic measures and behavioral and health-related factors at baseline were considered to account for potential confounding effects. In the BRHS, these covariates included age, occupational social class (manual/nonmanual) derived from the longest-held occupation, current smoker (yes/no), moderate to heavy alcohol consumption (yes/no), history of cardiovascular disease (CVD) or diabetes (yes/no), obesity (yes/no), and history of depression (yes/no). In Health ABC, the covariates included age, sex (male/female), ethnicity (White/African American), educational attainment (less than high school/high school graduate/postsecondary), history of CVD or diabetes (yes/no), obesity (yes/no), and history of depression (yes/no).

### Statistical analysis

Analyses were performed separately for the BRHS and Health ABC samples. Descriptive characteristics at baseline were presented as means and standard deviations for continuous variables or as percentages for categorical variables. Sankey diagrams were applied to present the transition of frailty status from baseline to follow-up. We considered the probability of both processes (ie, progressing to frailty and reversing from frailty according to social engagement and loneliness). Multinomial regression models were conducted to examine the associations of social connection (social engagement/loneliness) with progression to frailty and reversal from frailty. In the analysis of progression to frailty, samples included participants who were robust at both time points (sustained robust, which was the reference group), moved from robust to prefrail status (progression to prefrailty), and moved from robust/prefail to frail status (progression to frailty). For the reversal of frailty, the analytical sample included participants who were frail at both time points (sustained frail, which was the reference group) and those who improved their frailty status from frail to prefrail (reversal to prefrailty) and from frail/prefrail to robust (reversal to robust). For both analyses, categories of progression to frailty or reversal of frailty were dependent variables. Baseline levels of social engagement/loneliness, as well as change in social engagement/loneliness, were independent variables. All covariates were adjusted in the models. Supplementary analyses were undertaken comparing the cohort characteristics of both study samples when they entered the study (start of study) and at 2 time points, which forms the baseline and follow-up of the present analyses (presented in Table S4). All the analyses were conducted in SAS (version 9.4) and R (version 4.0.4).

## Results

**Figure 1:**
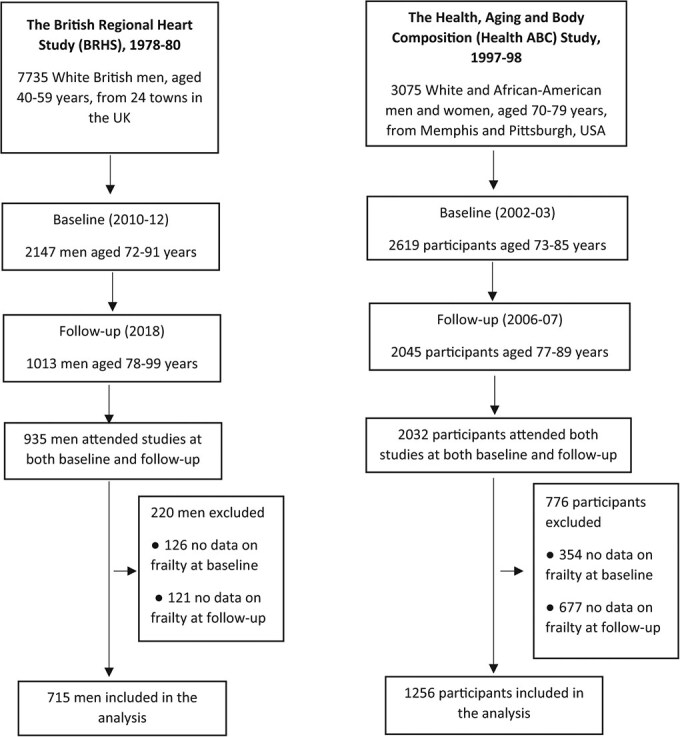
Profiles of the British Regional Heart Study (BRHS) and the Health, Aging and Body Composition (Health ABC) Study samples in this analysis.

The study sample in the BRHS consisted of 715 men (Figure 1). At baseline, the mean (SD) age of participants was 77.96 (3.75) years. In total, 376 participants (52.6%) were prefrail and 66 (9.2%) were frail. In Health ABC, 1256 were included in the analysis, of whom 656 (52.2%) were female and 403 (32.1%) were African American. The mean (SD) age of participants at baseline was 78.07 (2.77) years. A total of 756 (60.2%) participants were classified as robust, 467 (37.2%) as prefail, and 33 (2.6%) as frail. In both studies, the mean scores for social engagement at baseline were highest among the robust participants, followed by the prefrail, and were lowest among the frail participants. Mean scores for loneliness were highest among frail participants while lowest among robust participants. Other baseline characteristics of participants in the BRHS and Heath ABC are shown in Table 1.

**Table 1:** Baseline characteristics of participants in the BRHS (2010-2012) and Health ABC (2002-2003) included in this analysis.

**BRHS**				
	**Robust**	**Prefrail**	**Frail**	**Total**
All, *n* (%)	273 (38.2)	376 (52.6)	66 (9.2)	715 (100.0)
Social engagement, mean (SD)	4.73 (1.76)	4.40 (1.68)	4.29 (1.66)	4.52 (1.71)
Loneliness, mean (SD)	0.98 (1.25)	1.27 (1.51)	2.02 (1.79)	1.23 (1.47)
Age at baseline, mean (SD)	76.15 (3.13)	77.40 (3.95)	77.82 (4.32)	76.96 (3.75)
Social class group, *n* (%)				
Nonmanual	153 (56.0)	219 (58.2)	40 (60.6)	412 (57.6)
Manual	114 (41.8)	148 (39.4)	23 (34.9)	285 (39.9)
Missing	6 (2.2)	9 (2.4)	3 (4.6)	18 (2.5)
Current smoker, *n* (%)				
No	263 (96.3)	366 (97.3)	65 (98.5)	694 (97.1 )
Yes	8 (2.9)	9 (2.4)	1 (1.5)	18 (2.5)
Missing	2 (0.7)	1 (0.3)		3 (0.4)
Moderate to heavy alcohol consumption, *n* (%)				
No	259 (94.9)	355 (94.4)	66 (100.0)	680 (95.1)
Yes	12 (4.4)	18 (4.8)		30 (4.2)
Missing	2 (0.7)	3 (0.8)		5 (0.7)
History of CVD or diabetes, *n* (%)				
No	192 (70.3)	221 (58.8)	25 (37.9)	438 (61.3)
Yes	78 (28.6)	150 (39.9)	40 (60.6)	268 (37.5)
Missing	3 (1.1)	5 (1.3)	1 (1.5)	9 (1.3)
Obesity, *n* (%)				
No	234 (85.7)	303 (80.6)	47 (71.2)	584 (81.7)
Yes	39 (14.3)	73 (19.4)	19 (28.8)	131 (18.3)
History of depression, *n* (%)				
No	258 (94.5)	355 (89.1)	56 (84.9)	649 (90.8)
Yes	3 (1.1)	10 (2.7)		13 (1.8)
Missing	12 (4.4)	31 (8.2)	10 (15.2)	53 (7.4)
**Health ABC**				
	**Robust**	**Prefail**	**Frail**	**Total**
All, *n* (%)	756 (60.2)	467 (37.2)	33 (2.6)	1256 (100.0)
Social engagement, mean (SD)	6.85 (1.80)	6.33 (1.92)	5.58 (2.08)	6.62 (1.88)
Loneliness, mean (SD)	0.28 (0.57)	0.34 (0.66)	0.61 (0.66)	0.31 (0.61)
Age at baseline, mean (SD)	77.80 (2.66)	78.40 (2.87)	79.67 (2.81)	78.07 (2.77)
Sex, *n* (%)				
Male	361 (47.8)	223 (47.8)	16 (48.5)	600 (47.8)
Female	395 (52.3)	244 (52.3)	17 (51.5)	656 (52.2)
Ethnicity, *n* (%)				
White	540 (71.4)	291 (62.3)	22 (66.7)	853 (67.9)
African American	216 (28.6)	176 (37.7)	11 (33.3)	403 (32.1)
Education, *n* (%)				
Less than high school	121 (16.0)	98 (21.0)	12 (36.4)	231 (18.4)
High school graduate	240 (31.8)	136 (29.1)	9 (27.3)	385 (30.7)
Postsecondary	394 (52.1)	232 (19.7)	12 (36.4)	638 (50.8)
Missing	1 (0.1)	1 (0.2)		2 (0.2)
History of CVD or diabetes, *n* (%)				
No	562 (74.3)	324 (69.4)	23 (69.7)	909 (72.4)
Yes	194 (25.7)	143 (30.6)	10 (30.3)	347 (27.6)
Obesity, *n* (%)				
No	592 (78.3)	339 (72.6)	19 (57.6)	950 (75.6)
Yes	164 (21.7)	128 (27.4)	14 (42.4)	306 (24.4)
History of depression, *n* (%)				
No	618 (81.8)	339 (72.6)	15 (45.5)	972 (77.4)
Yes	138 (18.3)	128 (27.4)	18 (54.6)	284 (22.6)

### Transitions of frailty status in the BRHS and Health ABC

**Figure 2:**
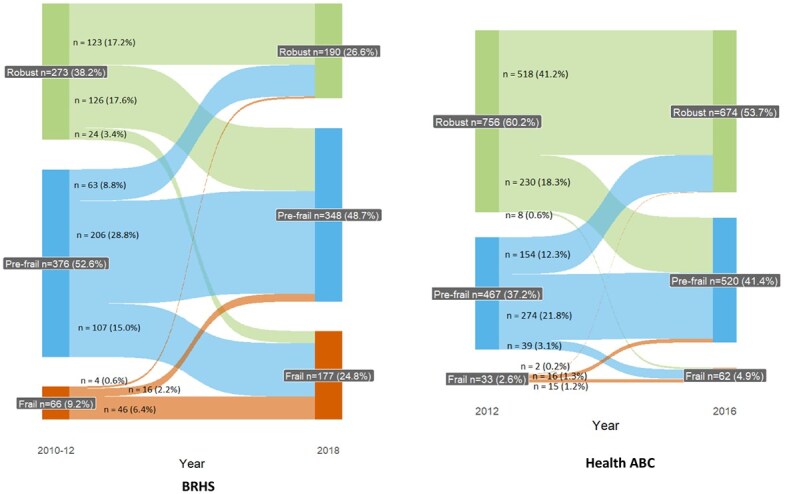
Changes in the frailty stages over time.

Figure 2 shows the dynamic change of frailty status over time in the BRHS and Health ABC. In the BRHS, 123 (17.2%) participants remained robust during the follow-up period. Progression to prefrail was observed in 126 (17.6%) robust participants, and 131 (18.3%) robust/prefrail participants developed frailty at follow-up. We also observed that 16 (2.2%) of participants improved their frailty status from frail to prefail and 67 (9.4%) from frail/prefrail to robust. A total of 46 (6.4%) participants were frail at both baseline and follow-up. In Health ABC, 230 (18.3%) robust participants became prefrail, and 47 (37.4%, 8 robust and 39 prefrail) participants became frail after 4 years of follow-up. In contrast, 154 (12.3%) prefrail participants reversed their status to robust, and 16 (1.3%) frail participants became prefrail. A total of 807 (64.0%) participants did not change frailty status, of whom 518 (41.2%) continued robust, 274 (21.8%) stayed prefail, and 15 (1.2%) remained frail.

### Associations of social engagement and loneliness with progression to prefrailty and frailty

Table 2 shows the relative risk ratios (RRRs) of progression to prefrailty and frailty, compared to sustained robustness, according to baseline and change in social engagement and loneliness. In the BRHS cohort, participants with a higher baseline score of social engagement had a decreased risk of becoming prefrail or frail. A 1-unit increase in the social engagement score at baseline was associated with a 21% (RRR, 0.79 [95% CI, 0.66-0.96]) lower risk of being prefrail relative to sustained robust and a 31% (RRR, 0.69 [95% CI, 0.55-0.85]) reduced risk of being frail (vs sustained robust). Participants who increased social engagement during the follow-up period also reduced their risk of becoming frail (RRR, 0.73 [95% CI, 0.59-0.90]). With respect to loneliness, a higher score at baseline (RRR, 1.42 [95% CI, 1.10-1.83]) and an increase in loneliness (RRR, 1.50 [95% CI, 1.18-1.90]) were associated with an elevated risk of developing frailty at follow-up.

**Table 2:** Relative risk ratios (RRRs) of progression to prefrailty and frailty according to baseline and change in social engagement and loneliness, compared with sustained robustness.

**BRHS** ^**a**^						
	**Progression to prefrailty vs sustained robustness**			**Progression to frailty vs sustained robustness**		
	**RRR**	**95% CI**	***P* value**	**RRR**	**95% CI**	***P* value**
Social engagement (*n* = 338)						
Baseline	0.79	(0.66-0.96)	.018	0.69	(0.55-0.85)	.0005
Change	0.87	(0.72-1.06)	.17	0.73	(0.59-0.90)	.0040
Loneliness (*n* = 323)						
Baseline	1.13	(0.88-1.46)	.34	1.42	(1.10-1.83)	.0074
Change	1.27	(1.01-1.59)	.044	1.50	(1.18-1.90)	.0009
**Health ABC** ^**b**^						
	**Progression to prefrailty vs sustained robust**			**Progression to frailty vs sustained robust**		
	**RRR**	**95% CI**	***P* value**	**RRR**	**95% CI**	***P* value**
Social engagement (*n* = 795)						
Baseline	0.92	(0.83-1.02)	.11	0.56	(0.45-0.70)	<.0001
Change	0.86	(0.77-0.97)	.013	0.51	(0.41-0.63)	<.0001
Loneliness (*n* = 787)						
Baseline	1.35	(0.95-1.92)	.089	1.74	(0.98-3.09)	.059
Change	1.27	(0.96-1.68)	.096	1.47	(0.91-2.39)	.12

^a^In BRHS, covariates including age, social class group, smoking, alcohol intake, history of CVD or diabetes, obesity, and history of depression were adjusted.

^b^In Health ABC, covariates including age, sex, ethnicity, educational attainment, history of CVD and diabetes, obesity, and history of depression were adjusted.

In Health ABC, there was a 44% relative risk reduction of becoming frail (vs being sustained robust) for each 1-unit increase in social engagement score at baseline (RRR, 0.56 [95% CI, 0.45-0.70]). In addition, an increase in social engagement during the follow-up was associated with a lower risk of developing prefrailty (RRR, 0.86 [95% CI, 0.77-0.97]) and frailty (RRR, 0.51 [95% CI, 0.41-0.63]). No significant association was found between loneliness (baseline or change in score) and progression to prefrailty and frailty in Health ABC.

### Associations of social engagement and loneliness with reversal to prefrailty and robust status

Table 3 presents the RRRs for participants who reversed their frail status to prefrail or robust status relative to those remaining frail, according to baseline and change in social engagement and loneliness scores. In the BRHS, prefrail and frail participants who increased their social engagement during follow-up were more likely to become robust (RRR, 1.71 [95% CI, 1.18-2.50]). For those who experienced a higher level of loneliness at baseline and increased loneliness during follow-up, their probability of reversal of their frailty status from frail to prefrail (baseline: RRR, 0.51 [95% CI, 0.29-0.91]; change: RRR, 0.49 [95% CI, 0.28-0.86]) and from frail/prefrail to robust (baseline: RRR, 0.37 [95% CI, 0.23-0.60]; change: RRR, 0.57 [95% CI, 0.37-0.87]) was roughly half as likely.

**Table 3:** Relative risk ratios (RRRs) of reversion to prefrailty and robust status according to baseline and change of social engagement and loneliness, compared with persistent frailty.

**BRHS** ^**a**^						
	**Reversal to prefrailty vs persistent frailty**			**Reversal to robust vs persistent frailty**		
	**RRR**	**95% CI**	***P* value**	**RRR**	**95% CI**	***P* value**
Social engagement (*n* = 123)						
Baseline	0.93	(0.58-1.50)	.78	1.38	(0.96-2.00)	.086
Change	1.34	(0.85-2.10)	.20	1.71	(1.18-2.50)	.0050
Loneliness (*n* = 118)						
Baseline	0.51	(0.29-0.91)	.021	0.37	(0.23-0.60)	<.0001
Change	0.49	(0.28-0.86)	.013	0.57	(0.37-0.87)	.0085
**Health ABC** ^**b**^						
	**Reversal to prefrailty vs persistent frailty**			**Reversal to robust vs persistent frailty**		
	**RRR**	**95% CI**	***P* value**	**RRR**	**95% CI**	***P* value**
Social engagement (*n* = 187)						
Baseline	1.50	(0.89-2.53)	.13	1.63	(1.08-2.47)	.020
Change	2.14	(1.24-3.68)	.0062	1.79	(1.17-2.74)	.0071
Loneliness (*n* = 187)						
Baseline	1.69	(0.45-6.40)	.44	0.76	(0.26-2.21)	.61
Change	1.40	(0.49-4.04)	.53	0.68	(0.28-1.63)	.39

^a^In BRHS, covariates including age, social class group, smoking, alcohol intake, history of CVD or diabetes, obesity, and history of depression were adjusted.

^b^In Heath ABC, covariates including age, sex, ethnicity, educational attainment, history of CVD and diabetes, obesity, and history of depression were adjusted.

In Heath ABC, we also observed beneficial effects of social engagement on improving frailty status. Increasing social engagement during follow-up significantly increased the probability of reversing frailty status from frail to prefrail (RRR, 2.14 [95% CI, 1.24-3.68]). In addition, participants who scored higher for social engagement at baseline (RRR, 1.63 [95% CI, 1.08-2.47]) or increased social engagement during follow-up (RRR, 1.79 [95% CI, 1.17-2.74]) were more likely to reverse their frailty status to robust. There were no significant effects of loneliness on reversal of frailty status.

## Discussion

This study examined frailty trajectories and their associations with both social engagement and loneliness among community-dwelling older adults from 2 longitudinal studies from the United Kingdom and the United States. We found that around 36% of the sample from the BRHS in the United Kingdom and 22% from Health ABC in the United States experienced worsening in frailty during follow-up of approximately 8 years and 4 years, respectively. Over the same time period, about 12% participants from the BRHS and 14% from Health ABC experienced an improvement in frailty status. These results indicate that although frailty is a distinctive health state related to the aging process, it is not an inevitable part of aging and is potentially reversible. Among individuals who experienced improvements in frailty, most (76% in BRHS; 90% in the Health ABC) moved from prefrail to robust status, which indicates that the potential for improvement is greater in the earlier stage of frailty development.

In both cohorts, we found baseline levels of and change in social engagement were associated independently with progression to frailty. This finding is consistent with previous studies and provides additional evidence that being socially active in later life could attenuate the risk of developing frailty.^26^^,^^31^^,^^36^ One explanation for this association is that individuals who are socially engaged and connected are more likely to have healthier behaviors, probably due to the influence of friends and loved ones who support them to adopt a healthy lifestyle. Besides, having multiple social ties provides more sources of information and thus increases the likelihood to receive wider support and access to appropriate health care.^37^^-^^41^ In the BRHS, we found that a higher score for loneliness at baseline predicted the risk of frailty over 8 years, which suggests that the deleterious effect of loneliness on physical frailty persists over time. In addition, an increase in loneliness can also elevate the risk of becoming frail. Feeling lonely is itself a stressor that can causes anxiety, depression, and hostility. Such negative effects and reactivity would promote chronic elevations in the physical system (eg, elevated vascular activation), increase delays in seeking care, and decrease medical compliance and health care utilization. Furthermore, loneliness can contribute to frailty through diminishing healthy behaviors such as poor nutrition, less exercise, and fragmented sleep.^18^^,^^42^^-^^45^

In addition to progression to frailty, this study also found significant effects of social engagement and loneliness on the reversal of frailty. For older adults who were already prefrail or frail, increased social engagement was associated with frailty reversal in both cohorts. This result further confirms the beneficial effects of social engagement in improving frailty among older adults.^46^ Moreover, we found that older adults in the BRHS who had a high level of loneliness at baseline and those who experienced an increase in loneliness were less likely to recover from frailty. These associations were observed in both studies of community-dwelling older adults in the United States and United Kingdom, providing some consistency and robustness to the findings. Despite cultural differences and differences in terms of health care, the results were mostly consistent in both study populations. Collectively, our study findings point to the importance of social engagement in preventing and improving frailty among older adults.

The strengths and limitations of this study need to be considered. A key strength of this study is the assessment of prospective associations of both quantitative and qualitative aspects of social connections with frailty through measures of both social engagement and loneliness in 2 distinct population cohort studies. Undertaking epidemiologic investigations in these 2 cohorts helps to assess consistency (or reproducibility), which is a key criterion to assess associations in epidemiologic studies. In testing the association in the 2 study samples, we also provide findings on longitudinal associations between social engagement and frailty, using valid and reliable measures of exposures and outcomes. The measures of social engagement and frailty used are the same in the 2 studies. Another strength is the investigation of the dynamic nature of social connections and frailty, as well as changes in these measures over time. A potential limitation of this study is the generalizability of the findings. The design features of the cohorts meant that the BRHS consisted of White British men only, and Health ABC recruited White and African American men and women living in only 2 areas (Pittsburgh and Memphis) in the United States. Future research using larger population-based studies, particularly with greater representation from women and other ethnic minorities, is needed to better understand the association between social connections and frailty among diverse populations of older adults. Besides, like many longitudinal cohort studies of older adults, this study inevitably suffered from survival bias. Participants who were younger and healthier were more likely to attend the follow-up of the studies. In the BRHS, participants in the present analyses, compared to those who withdrew or died before our study period, were younger and healthier. A similar pattern was observed in the Health ABC Study (Table S4). Although survival bias was inevitable, these cohort studies of older adults offered the opportunity to examine the role of social connections in frailty among older age. This survival bias, if anything, might have led to a slight underestimation of the association between social connections and frailty, as surviving participants tended to be healthier. In Health ABC, loneliness was measured by a single-item question, which may have limited content validity and sensitivity. This may explain the absence of an association between loneliness and frailty in that cohort. Furthermore, although our studies attempted to adjust for several confounders, information on some factors (eg, smoking and alcohol used) in the Health ABC was unavailable. The possibility of residual confounding cannot be totally excluded. Additionally, previous studies have suggested that the relationship between social connections and frailty could be bidirectional.^13^^,^^30^ Although our study was longitudinal in design and found that social connections at baseline were associated with frailty at follow-up, which could potentially support causal relationships, the possibility that frailty, conversely, could influence social connections was not tested in this study. Future longitudinal studies examining these associations in both directions would strengthen our understanding about the links between social connections and frailty in older adults.

Frailty has been recognized as an emerging public health issue among older adults.^47^ Campaigns across countries have raised awareness to reduce the burden of frailty.^48^^-^^51^ Notably, the National Health Service in England has introduced routine frailty identification for patients aged 65 years and older registered in the General Practice (GP) system.^52^ To date, interventions on physical exercise and nutrition have been shown to be the most effective in improving frailty.^52^^-^^54^ Although an increasing number of studies highlight the potentially important role of social connections in frailty, intervention studies targeting these issues are limited.^13^ The issues of social isolation and loneliness have not been given sufficient attention in intervention studies, strategies, or action plans for preventing frailty in older adults. Our findings, which show that social engagement and loneliness were associated with the progression as well as reversal of frailty, suggest that it could be potentially important for health and social care professionals to consider assessing social ties, social activities, and perceived loneliness along with identification of frailty risk. Population-based intervention strategies such as enhancing social connections and building age-friendly communities that provide opportunities for social interactions among older adults could contribute to reducing the burden of frailty.

Although frailty is a common condition in aging populations, development of frailty is manageable, preventable, and potentially reversible. This study provides evidence that social inactivity and loneliness are potentially important factors that increase the risk of developing frailty as well as hinder its reversal. Increasing social engagement and reducing loneliness among older adults could be beneficial in reducing the burden of frailty. Findings from this study, together with other related studies, highlight the importance of considering social connections as a crucial and modifiable factor in interventions to promote healthy aging. Further observational and intervention studies are needed to examine this further.

## Supplementary Material

Web_Material_kwae221

## Data Availability

Data for the British Regional Heart Study (BRHS) are available from https://www.ucl.ac.uk/epidemiology-health-care/research/primary-care-and-population-health/research/brhs. Data for the Health, Aging and Body Composition (Health ABC) Study are available from https://healthabc.nia.nih.gov/.

## References

[1] Clegg A , Young J, Iliffe S, et al. Frailty in elderly people. *Lancet*. 2013;381(9868):752-762. 10.1016/S0140-6736(12)62167-9 23395245 PMC4098658

[2] Collard RM , Boter H, Schoevers RA, et al. Prevalence of frailty in community-dwelling older persons: a systematic review. *J Am Geriatr Soc*. 2012;60(8):1487-1492. 10.1111/j.1532-5415.2012.04054.x 22881367

[3] Kojima G . Frailty as a predictor of disabilities among community-dwelling older people: a systematic review and meta-analysis. *Disabil Rehabil*. 2017;39(19):1897-1908. 10.1080/09638288.2016.1212282 27558741

[4] Kojima G , Iliffe S, Walters K. Frailty index as a predictor of mortality: a systematic review and meta-analysis. *Age Ageing*. 2018;47(2):193-200. 10.1093/ageing/afx162 29040347

[5] Kojima G . Frailty as a predictor of future falls among community-dwelling older people: a systematic review and meta-analysis. *J Am Med Dir Assoc*. 2015;16(12):1027-1033. 10.1016/j.jamda.2015.06.018 26255098

[6] Kojima G . Frailty as a predictor of hospitalisation among community-dwelling older people: a systematic review and meta-analysis. *J Epidemiol Community Health*. 2016;70(7):722-729. 10.1136/jech-2015-206978 26933121

[7] Kojima G . Frailty as a predictor of nursing home placement among community-dwelling older adults: a systematic review and meta-analysis. *J Geriatr Phys Ther*. 2018;41(1):42-48. 10.1519/JPT.0000000000000097 27341327

[8] Buckinx F , Rolland Y, Reginster J-Y, et al. Burden of frailty in the elderly population: perspectives for a public health challenge. *Arch Public Health*. 2015;73(1):1-7. 10.1186/s13690-015-0068-x 25866625 PMC4392630

[9] Jarach CM , Tettamanti M, Nobili A, et al. Social isolation and loneliness as related to progression and reversion of frailty in the Survey of Health Aging Retirement in Europe (SHARE). *Age Ageing*. 2021;50(1):258-262. 10.1093/ageing/afaa168 32915990 PMC7793602

[10] Fried LP , Tangen CM, Walston J, et al. Frailty in older adults: evidence for a phenotype. *J Gerontol A Biol Sci Med Sci*. 2001;56(3):M146-M157. 10.1093/gerona/56.3.M146 11253156

[11] Mitnitski AB , Mogilner AJ, Rockwood K. Accumulation of deficits as a proxy measure of aging. *Scientific World Journal*. 2001;1(2):323-336. 10.1100/tsw.2001.58 12806071 PMC6084020

[12] Cesari M , Gambassi G, Abellan van Kan G, et al. The frailty phenotype and the frailty index: different instruments for different purposes. *Age Ageing*. 2014;43(1):10-12. 10.1093/ageing/aft160 24132852

[13] Mehrabi F , Beland F. Effects of social isolation, loneliness and frailty on health outcomes and their possible mediators and moderators in community-dwelling older adults: a scoping review. *Arch Gerontol Geriatr*. 2020;90:104119. 10.1016/j.archger.2020.104119 32562956

[14] Bennett KM . Low level social engagement as a precursor of mortality among people in later life. *Age Ageing*. 2002;31(3):165-168. 10.1093/ageing/31.3.165 12006303

[15] Berkman LF , Glass T, Brissette I, et al. From social integration to health: Durkheim in the new millennium. *Soc Sci Med*. 2000;51(6):843-857. 10.1016/S0277-9536(00)00065-4 10972429

[16] Meltzer H , Bebbington P, Dennis MS, et al. Feelings of loneliness among adults with mental disorder. *Soc Psychiatry Psychiatr Epidemiol*. 2013;48(1):5-13. 10.1007/s00127-012-0515-8 22570258

[17] Cacioppo JT , Cacioppo S. Social relationships and health: the toxic effects of perceived social isolation. *Soc Personal Psychol Compass*. 2014;8(2):58-72. 10.1111/spc3.12087 24839458 PMC4021390

[18] Hawkley LC , Cacioppo JT. Loneliness matters: a theoretical and empirical review of consequences and mechanisms. *Ann Behav Med*. 2010;40(2):218-227. 10.1007/s12160-010-9210-8 20652462 PMC3874845

[19] Valtorta NK , Kanaan M, Gilbody S, et al. Loneliness, social isolation and risk of cardiovascular disease in the English Longitudinal Study of Ageing. *Eur J Prev Cardiol*. 2018;25(13):1387-1396. 10.1177/2047487318792696 30068233

[20] Deckx L , van den Akker M, Buntinx F. Risk factors for loneliness in patients with cancer: a systematic literature review and meta-analysis. *Eur J Oncol Nurs*. 2014;18(5):466-477. 10.1016/j.ejon.2014.05.002 24993076

[21] Kraav S-L , Awoyemi O, Junttila N, et al. The effects of loneliness and social isolation on all-cause, injury, cancer, and CVD mortality in a cohort of middle-aged Finnish men. A prospective study. *Aging Ment Health*. 2021;25(12):2219-2228. 10.1080/13607863.2020.1830945 33939562

[22] Samtani S , Mahalingam G, Lam BCP, et al. Associations between social connections and cognition: a global collaborative individual participant data meta-analysis. *Lancet Healthy Longevity.* 2022;3(11):e740-e753. 10.1016/S2666-7568(22)00199-4 36273484 PMC9750173

[23] Janke MC , Payne LL, Van Puymbroeck M. The role of informal and formal leisure activities in the disablement process. *Int J Aging Hum Dev*. 2008;67(3):231-257. 10.2190/AG.67.3.c 19049245

[24] Kuiper JS , Zuidersma M, Voshaar RCO, et al. Social relationships and risk of dementia: a systematic review and meta-analysis of longitudinal cohort studies. *Ageing Res Rev*. 2015;22:39-57. 10.1016/j.arr.2015.04.006 25956016

[25] Kraav S-L , Lehto SM, Kauhanen J, et al. Loneliness and social isolation increase cancer incidence in a cohort of Finnish middle-aged men. A longitudinal study. *Psychiatry Res*. 2021;299:113868. 10.1016/j.psychres.2021.113868 33774371

[26] Davies K , Maharani A, Chandola T, et al. The longitudinal relationship between loneliness, social isolation, and frailty in older adults in England: a prospective analysis. *Lancet Healthy Longevity*. 2021;2(2):e70-e77. 10.1016/S2666-7568(20)30038-6 36098160

[27] Han SY , Jang HY, Ko Y. Factors influencing the stages of frailty among Korean older adults focusing on objective and subjective social isolation. *BMC Geriatr*. 2022;22(1):488. 10.1186/s12877-022-03179-0 35672657 PMC9175502

[28] Herrera-Badilla A , Navarrete-Reyes AP, Amieva H, et al. Loneliness is associated with frailty in community-dwelling elderly adults. *J Am Geriatr Soc*. 2015;63(3):607-609. 10.1111/jgs.13308 25800917

[29] Hoogendijk EO , Suanet B, Dent E, et al. Adverse effects of frailty on social functioning in older adults: results from the Longitudinal Aging Study Amsterdam. *Maturitas*. 2016;83:45-50. 10.1016/j.maturitas.2015.09.002 26428078

[30] Gale CR , Westbury L, Cooper C. Social isolation and loneliness as risk factors for the progression of frailty: the English Longitudinal Study of Ageing. *Age Ageing*. 2018;47(3):392-397. 10.1093/ageing/afx188 29309502 PMC5920346

[31] Etman A , Kamphuis CB, Van der Cammen TJ, et al. Do lifestyle, health and social participation mediate educational inequalities in frailty worsening? *Eur J Public Health* . 2015;25(2):345-350. 10.1093/eurpub/cku093 25061232 PMC4447813

[32] Sun J , Kong X, Li H, et al. Does social participation decrease the risk of frailty? Impacts of diversity in frequency and types of social participation on frailty in middle-aged and older populations. *BMC Geriatr*. 2022;22(1):553. 10.1186/s12877-022-03219-9 35778684 PMC9250233

[33] Walker M , Whincup P, Shaper A. The British regional heart study 1975–2004. *Int J Epidemiol*. 2004;33(6):1185-1192. 10.1093/ije/dyh295 15319395

[34] Fredman L , Cauley JA, Satterfield S, et al. Caregiving, mortality, and mobility decline: the health, aging, and body composition (Health ABC) study. *Arch Intern Med*. 2008;168(19):2154-2162. 10.1001/archinte.168.19.2154 18955646 PMC3260883

[35] Kimble R , McLellan G, Lennon LT, et al. Association between oral health markers and decline in muscle strength and physical performance in later life: longitudinal analyses of two prospective cohorts from the UK and the USA. *Lancet Healthy Longevity.* 2022;3(11):e777-e788. 10.1016/S2666-7568(22)00222-7 36356627 PMC10397540

[36] Ge L , Yap CW, Heng BH. Associations of social isolation, social participation, and loneliness with frailty in older adults in Singapore: a panel data analysis. *BMC Geriatr*. 2022;22(1):1-10. 10.1186/s12877-021-02745-2 34991493 PMC8734362

[37] Cohen S. Psychosocial stress, social networks and susceptibility to infection. In: Koenig HG, Cohen HJ, eds. The Link Between Religion and Health: Psychoneuroimmunology and the Faith Factor. Oxford University Press; 2002:101–123.

[38] Scheffler RM , Brown TT. Social capital, economics, and health: new evidence. *Health Econ Policy Law*. 2008;3(4):321-331. 10.1017/S1744133108004593 18793475

[39] Coleman JS . Social capital in the creation of human capital. *Am J Sociol*. 1988;94:S95-S120. 10.1086/228943

[40] Ferlander S . The importance of different forms of social capital for health. *Acta Sociol*. 2007;50(2):115-128. 10.1177/0001699307077654

[41] Nieminen T , Prättälä R, Martelin T, et al. Social capital, health behaviours and health: a population-based associational study. *BMC Public Health*. 2013;13(1):1-11. 10.1186/1471-2458-13-613 23805881 PMC3722011

[42] Yannakoulia M , Ntanasi E, Anastasiou CA, et al. Frailty and nutrition: from epidemiological and clinical evidence to potential mechanisms. *Metabolism*. 2017;68:64-76. 10.1016/j.metabol.2016.12.005 28183454

[43] Theou O , Stathokostas L, Roland KP, et al. The effectiveness of exercise interventions for the management of frailty: a systematic review. *J Aging Res*. 2011;2011:1-19. 10.4061/2011/569194 PMC3092602 21584244

[44] Pourmotabbed A , Boozari B, Babaei A, et al. Sleep and frailty risk: a systematic review and meta-analysis. *Sleep Breathing*. 2020;24(3):1187-1197. 10.1007/s11325-020-02061-w 32215833

[45] Cacioppo JT , Hawkley LC. Social isolation and health, with an emphasis on underlying mechanisms. *Perspect Biol Med*. 2003;46(3):S39-S52. 10.1353/pbm.2003.0049 14563073

[46] Heaven B , Brown LJ, White M, et al. Supporting well-being in retirement through meaningful social roles: systematic review of intervention studies. *Milbank Q*. 2013;91(2):222-287. 10.1111/milq.12013 23758511 PMC3696198

[47] Cesari M , Prince M, Thiyagarajan JA, et al. Frailty: an emerging public health priority. *J Am Med Dir Assoc*. 2016;17(3):188-192. 10.1016/j.jamda.2015.12.016 26805753

[48] Canadian Frailty Network . Canadian Frailty Network Avoide Frailty Program for Health Aging. 2012. https://www.cfn-nce.ca/

[49] NHS . The NHS long term plan. 2019. https://www.longtermplan.nhs.uk/

[50] Shinkai S , Yoshida H, Taniguchi Y, et al. Public health approach to preventing frailty in the community and its effect on healthy aging in Japan. *Geriatr Gerontol Int*. 2016;16(suppl 1):87-97. 10.1111/ggi.12726 27018287

[51] Woo J . Designing fit for purpose health and social services for ageing populations. *Int J Environ Res Public Health*. 2017;14(5):457. 10.3390/ijerph14050457 28441324 PMC5451908

[52] Negm AM , Kennedy CC, Thabane L, et al. Management of frailty: a systematic review and network meta-analysis of randomized controlled trials. *J Am Med Dir Assoc*. 2019;20(10):1190-1198. 10.1016/j.jamda.2019.08.009 31564464

[53] Hsieh T-J , Su S-C, Chen C-W, et al. Individualized home-based exercise and nutrition interventions improve frailty in older adults: a randomized controlled trial. *Int J Behav Nutr Phys Act*. 2019;16(1):1-15. 10.1186/s12966-019-0855-9 31791364 PMC6889427

[54] Liao C-D , Lee P-H, Hsiao D-J, et al. Effects of protein supplementation combined with exercise intervention on frailty indices, body composition, and physical function in frail older adults. *Nutrients*. 2018;10(12):1916. 10.3390/nu10121916 30518122 PMC6315527

