# Supplementary material for: Associations of social engagement and loneliness with the progression and reversal of frailty: longitudinal investigations of 2 prospective cohorts from the UK and the USA
Source: Am J Epidemiol. 2024 Jul 25;194(4):984–93. doi: 10.1093/aje/kwae221 (PMC11978614; doi:10.1093/aje/kwae221)
Supplement: Web_Material_kwae221 [file web_material_kwae221.zip › Supplementary tables_Frailty social connections_AJE_150524.docx]

**Title: Associations of social engagement, and loneliness with the progression and reversal of frailty: longitudinal investigations of two prospective cohorts from the UK and the USA**

Table S1 – Table S4

**Table S1 Operationalization in this study of frailty according to the Fried frailty phenotype**

| **Criterion (Yes/No)** | **BRHS** | | **Health ABC** | |
| --- | --- | --- | --- | --- |
|  | Response item/Functional  measure | Note | Response item/functional  Measure | Note |
| 1. Unintentional weight loss | ● >= 5% unintentional weight loss from previous questionnaire/assessment | Specified not trying to lose weight or no change/unknown change in weight  but a substantial measured weight loss (i.e. >= 5%) since the  previous assessment. If  missing and self-reported weight loss of more than 7 pounds (3 kg) in past 3 months, then coded as unintentional weight loss | ● >= 5% unintentional weight loss  from previous assessment | Specified not trying to lose weight or no change/unknown change in weight in the questionnaire but a substantial measured  weight loss (i.e. >= 5%) since the previous assessment coded as  unintentional weight loss |
| 2. Fatigue | ● Do you feel full of energy? | Answering “no” coded as fatigue | ● “Please  describe your usual energy level in the past month, where 0 is no energy and 10 is the most energy | Energy level <=3. Where self-reported energy level was unavailable, self-reported feeling usually tired during the day was coded as fatigue. |
| 3. Low physical activity | ● Compared with a man who spends 2 hours on most days on activities such as: walking,  gardening, household chores, DIY projects, how physically active would you consider  yourself?” | Answering “much less active” coded as low physical activity.  If missing, information on self-reported no walking, cycling and  sporting physical exercise was  used to determine low activity level | ● kcal/wk spent on commonly performed physical activities:  walking, climbing stairs, and doing major chores calculated from a modified leisure-time  physical activity  questionnaire. | Lowest quintile stratified by sex  coded as low physical activity |
| 4. Weakness | ● Grip strength (Jamar Hydraulic Hand Dynamometer Model  J00105) highest of 3 readings in both hands. | Lowest quintile coded as weak.  Where measured grip  strength was unavailable, self-reported weak grip strength or inability to grip with hands (e.g. opening a jam jar) was  coded as weakness. | ● Grip strength (Jamar isometric dynamometer JLW Instruments, Chicago) highest of 2 readings in both hands. | Lowest quintile stratified by sex coded as weak. Where measured grip strength was unavailable due to pain or surgery on both hands, self-reported inability or a lot of  difficulty to grip with hands, or using your fingers to grasp or  handle was coded as weak. |
| 5. Slow walking speed | ● Gait speed (m/s) based on the time required to walk 3 m at normal pace. | Lowest quintile coded as slow walking speed. Where measured gait speed was unavailable, self-report of low walking pace (or being unable to walk more than a few steps, or <200 yards (approximately 180 m), or difficulty walking across a room) was used to determine slowness. | ● Gait speed (m/s) based on the  time required to walk 20 m at usual pace. | Lowest quintile stratified by sex coded as slow walking speed.  Where measured gait speed was unavailable, self-report of great difficulty or inability  to walk 0.25 mile was used to determine slowness. |

**Table S2 Calculation of social engagement score in this study**

| **Social engagement** | **BRHS** | | **Health ABC** | |
| --- | --- | --- | --- | --- |
|  | Items | Note | Items | Note |
| 1. Spending time with family, friends and neighbours  Score: 0(No) /1 (Yes) | ● Spending time with family, friends and neighbours  ● Talking with friends/relatives on the telephone in a typical week | Answering “Yes” in either of the items coded as 1 (yes).  Answering “No” in both items coded as 0 (No) | ● In a typical week, how often do you get together with friends or neighbours?  ● In a typical week, how often do you get together with your children or other relatives? | Answering “less than once a week” in both items coded as 0; otherwise coded as 1 |
| 2. Doing paid work  Score: 0(No) /1 (Yes) | ● Spending time in paid work | Answering “Yes” coded as 1 | ● Do you currently work for pay, either at a regular job, consulting, or doing odd jobs? | Answering “Yes” coded as 1; otherwise coded as 0 |
| 3. Doing voluntary work  Score: 0(No) /1 (Yes) | ● Spending time in voluntary work | Answering “Yes” coded as 1 | ● Do you currently do any volunteer work? | Answering “Yes” coded as 1; otherwise coded as 0 |
| 4. Playing cards, games, or bingo  Score: 0(No) /1 (Yes) | ● Spending time in playing cards, games, or bingo | Answering “Yes” coded as 1 | ● In the past 12 months,  how often did you Play board games, bingo, bridge,  or other card games? | Answering “not at all” coded as 0; otherwise coded as 1 |
| 5. Participating in religious activities  or social clubs  Score: 0(No) /1 (Yes) | ● Attending religious services in a typical week  ● Going to a pub or club | Answering “Yes” coded as 1 | ● In the past 12 months, how often did you participate in church, community, or social club activities? | Answering “not at all” coded as 0; otherwise coded as 1 |
| 6. Going on holidays or overnight trips  Score: 0(No) /1 (Yes) | ● Do you go on day or overnight trips?  ● Have you been on holiday in the last year? | Answering “Yes” in either of the items coded as 1 | ● In the past 12 months, how often did you travel 100 miles or more from your home? | Answering “not at all” coded as 0; otherwise coded as 1 |
| 7. Reading books or newspapers  Score: 0(No) /1 (Yes) | ● Spending time in reading | Answering “Yes” coded as 1 | ● In the past 12 months, how often did you read a newspaper or magazine article?  ● In the past 12 months, how often did you read a novel or non-fiction book, such as a biography? | Answering “not at all” in both items coded as 0; otherwise coded as 1 |
| 8. Using internet or writing letter  Score: 0(No) /1 (Yes) | ● Do you use the internet and or email? | Answering “Yes” coded as 1 | ● In the past 12 months, how often did you write a letter, e-mail, article, poem,or story? | Answering “not at all” coded as 0; otherwise coded as 1 |
| 9. Attending courses or public meetings  Score: 0(No) /1 (Yes) | ● Attending class or course of study | Answering “Yes” coded as 1 | ● In the past 12 months, how often did you take a class or adult education course?  ● In the past 12 months, how often did you attend a lecture, discussion, or public meeting? | Answering “not at all” in both items coded as 0; otherwise coded as 1 |
| 10. Eating out in the restaurants or visiting the cinema/sporting events/concert/museum/zoo/aquarium/science centre  Score: 0(No) /1 (Yes) | ● Visiting the cinema/restaurants/sporting events | Answering “Yes” coded as 1 | ● In the past 12 months, how often did you go out to a movie; attend a concert, the theatre, or a sports event; or visit a museum, zoo, aquarium, or science centre | Answering “not at all” coded as 0; otherwise coded as 1 |

Note. Total score of social engagement was calculating by summing up the scores from 10 dimensions. For both BRHS and Health ABC, the total scores range from 0-10.

**Table S3 Calculation of loneliness score in this study**

| **Loneliness** | | | |
| --- | --- | --- | --- |
| **BRHS** | | **Health ABC** | |
| Items | Note | Items | Note |
| ● How often do you feel you lack companionship?  Score: 0-2 | Answering “Hardly ever/Never” coded as 0, “Sometimes” as 1, “Often” as 2 | ● I felt lonely  Score: 0-3 | Answering “ Rarely/None of the time” coded as 0, “Some of the time” as 1, “Much of time” as 2, “Most or all of the time” as 3 |
| ● How often do you feel isolated from others?  Score: 0-2 | Answering “Hardly ever/Never” coded as 0, “Sometimes” as 1, “Often” as 2 |  |  |
| ● How often do you feel left out?  Score: 0-2 | Answering “Hardly ever/Never” coded as 0, “Sometimes” as 1, “Often” as 2 |  |  |
| ● How often do you feel in tune with the people around you?  Score: 0-2 | Answering “Often” coded as 0, “Sometimes” as 1, “Hardly ever/Never” as 2 |  |  |

Note. For the BRHS, total score of loneliness was calculating by summing up all the items, which ranges from 0-8. For Health ABC, the total score of loneliness ranges from 0-3.

**Table S4 Logistic regression for assessing selective survival bias based on key covariates in BRHS and Health ABC.**

Table S4.1: Odd ratios of characteristics of participants who took part in the study waves of 2010-2012 and 2014 in BRHS, compared with non-attenders

|  | OR | 95% CI |
| --- | --- | --- |
| **Age** | 0.81 | (0.79, 0.82) |
| **Social class group** (ref: non-manual) |  |  |
| Manual | 0.58 | (0.49, 0.69) |
| **Current smoker** (ref: no) |  |  |
| Yes | 0.35 | (0.29, 0.44) |
| **Moderate to heavy alcohol consumption** (ref: no) |  |  |
| Yes | 0.66 | (0.54, 0.79) |
| **History of CVD or diabetes** (ref: no) |  |  |
| Yes | 0.30 | (0.13, 0.58) |
| **Obesity** (ref: no) |  |  |
| Yes | 0.42 | (0.27, 0.64) |

Note: characteristics for all participants measured in wave 1 (1970-80) were used. Measure of depression was not available in the first wave.

Table S4.2: Odd ratios of characteristics of participants who took part in the study waves of 2002-03 and 2006-07 in Health ABC, compared with non-attenders

|  | OR | 95% CI |
| --- | --- | --- |
| **Age** | 0.90 | (0.88, 0.92) |
| **Sex** (ref: male) |  |  |
| Female | 1.09 | (0.93, 1.27) |
| **Ethnicity** (ref: white) |  |  |
| African American | 0.57 | (0.48, 0.68) |
| **Education** (ref: less than high school) |  |  |
| High school graduate | 1.20 | (0.97, 1.49) |
| Post-secondary | 1.73 | (1.41, 2.14) |
| **History of CVD or diabetes** (ref: no) |  |  |
| Yes | 0.70 | (0.60, 0.81) |
| **Obesity** (ref: no) |  |  |
| Yes | 0.97 | (0.81, 1.17) |
| **Depression** (ref: no) |  |  |
| Yes | 0.42 | (0.29, 0.60) |

Note: characteristics for all participants measured in wave 1 (1997-98) were used.
